# Aqueous Extract of Cocoa Phenolic Compounds Protects Differentiated Neuroblastoma SH-SY5Y Cells from Oxidative Stress

**DOI:** 10.3390/biom11091266

**Published:** 2021-08-25

**Authors:** Noelia Carballeda Sangiao, Susana Chamorro, Sonia de Pascual-Teresa, Luis Goya

**Affiliations:** 1 Departamento de Metabolismo y Nutrición, Instituto de Ciencia y Tecnología de Alimentos y Nutrición (ICTAN-CSIC), C/José Antonio Nováis, 10, 28040 Madrid, Spain; n.carballeda@ictan.csic.es; 2 Departamento de Genética, Fisiología y Microbiología, Facultad de Ciencias Biológicas, Universidad Complutense de Madrid (UCM), 28040 Madrid, Spain; schamorr@ucm.es

**Keywords:** epicatechin, neuronal-like cells, reactive oxygen species, glutathione, antioxidant defenses

## Abstract

Cocoa is a rich source of polyphenols, especially flavanols and procyanidin oligomers, with antioxidant properties, providing protection against oxidation and nitration. Cocoa phenolic compounds are usually extracted with methanol/ethanol solvents in order to obtain most of their bioactive compounds; however, aqueous extraction seems more representative of the physiological conditions. In this study, an aqueous extract of cocoa powder has been prepared and chemically characterized, and its potential protective effect against chemically-induced oxidative stress has been tested in differentiated human neuroblastoma SH-SY5Y cells. Neuronal-like cultured cells were pretreated with realistic concentrations of cocoa extract and its major monomeric flavanol component, epicatechin, and then submitted to oxidative stress induced by a potent pro-oxidant. After one hour, production of reactive oxygen species was evaluated by two different methods, flow cytometry and in situ fluorescence by a microplate reader. Simultaneously, reduced glutathione and antioxidant defense enzymes glutathione peroxidase and glutathione reductase were determined and the results used for a comparative analysis of both ROS (reactive oxygen species) methods and to test the chemo-protective effect of the bioactive products on neuronal-like cells. The results of this approach, never tested before, validate both analysis of ROS and indicate that concentrations of an aqueous extract of cocoa phenolics and epicatechin within a physiological range confer a significant protection against oxidative insult to neuronal-like cells in culture.

## 1. Introduction

Cognitive abilities are progressively deteriorated throughout the adult lifespan [1]. Genetic, endogenous, and environmental factors determine the decline of memory, attention, execution, and processing speed, aging being the greatest risk factor for cognitive deterioration and dementia [2]. Although the etiology of these processes is not well understood, it is presumed that it is related to an increase in oxidative stress and a reduction in the function of the immune system [3].

Increasing data suggest that lifestyle approaches, such as healthy dietary patterns and physical activity, can improve cognitive abilities and brain health throughout the human lifespan [4,5,6], as well as delay or prevent progressive cognitive decline [7,8]. Along this line, the study of plant-based dietary patterns and polyphenol-rich plant foods on either preventing or improving cognitive function has become an emergent area of research [9,10,11,12,13].

Cocoa is one of the best-known sources of dietary polyphenols, mainly monomeric flavanols such as epicatechin and catechin, as well as the dimers procyanidins B1 and B2 [14]. In recent years, several human clinical studies have concluded that consumption of cocoa and cocoa-derived products can be an effective means to improve general cognition and working memory, particularly among older populations at risk or with cognitive decline [3,12,15,16,17,18]. Most research on cocoa flavonoids as neuroprotective agents has been focused on brain endothelium and cerebro-vascular circulation [17,18,19,20,21]. In fact, in the past decade some studies showed that cocoa flavanols, especially epicatechin, directly affect the endothelial cells of brain vessels, stimulating activity of endothelial nitric oxide synthase (eNOS) and generating nitric oxide (NO); this increased NO induces vasodilation and improves cerebrovascular perfusion [3,15,18]. Similarly, confirmation that polyphenols, particularly cocoa flavanols, can journey through the blood-brain barrier towards neuronal protection [22,23] has encouraged the study of their effects on neuronal cell function, including their antioxidant capacity [24,25] and regulation of specific signaling pathways [3,7]. However, a comprehensive study of their chemo-protective effect by regulating the antioxidant defense system of neuronal-like cells in response to oxidative stress has not been addressed.

Together with positive regulation of the enzymatic and non-enzymatic antioxidant defense system, chemo-protective capacity involves quenching overproduction of reactive oxygen species (ROS) induced by oxidative stress. Analysis of ROS in plated cells with a dichlorofluorescein (H2DCFDA) fluorescent probe in a microplate reader has been widely used as a reliable method to evaluate cell ROS concentration [26,27]. Nevertheless, this method has been recently questioned and a flow cytometry approach has been recommended as a reliable alternative [25,28,29,30]. In order to unravel the specific changes in the cellular redox status prior to the study in experimental animals and humans, a reliable cell culture model needs to be established and the comprehensive response of its antioxidant defense system to stressful conditions finely characterized. Fully differentiated SH-SY5Y neurons provide a closer approximation of mature human neurons found in vivo than do their undifferentiated progenitor cell counterparts, providing an advantageous model for chemotherapeutic toxicity in neurons [31].

Thus, the aims of the present study were to show the potential chemo-protective effect of cocoa flavonoids against induced oxidative stress in cultured neuronal-like cells as well as to compare and validate two different methodologies for ROS determination in the same cell model and conditions.

## 2. Materials and Methods

### 2.1. Chemicals

All chemicals used were of high performance liquid chromatography (HPLC)-analytical grade. Epicatechin, quercetin-3-*O*-glucoside, l-phenylalanine, l-tryptophan, theobromine, caffeine, and tetramethylpirazine were purchased from Sigma Chemical Co (St. Louis, MO, USA).

### 2.2. Test Product and Polyphenol Extraction

A commercial semi-defatted cocoa powder from organic farming was kindly supplied by Salengei^®^ (Barcelona, Spain). It was labeled as sugar, sweetener, and emulsifier free and as containing 7000 mg of flavanols, 2500 mg of theobromine, and 100 mg of caffeine in 100 g of cocoa powder. For the extraction of the phenolic compounds, 5 g of the sample was placed in a capped centrifuge tube and suspended in 20 mL of distilled water, vortexed and sonicated over 15 min. Samples were then centrifuged at 5000 rpm for 10 min at 4 °C, and the supernatant was collected. The residue was re-suspended in 20 mL of water and re-extracted following the same procedure twice. The supernatants were combined and freeze dried until use. The water soluble extract was then used for cell culture assays. For the polyphenolic characterization, this water soluble cocoa extract was re-suspended in methanol/H_2_O (50:50 *v*/*v* acidified with formic acid 0.1%), filtered (0.45 μm), and placed in vials for subsequent HPLC coupled to Quadrupole Time-of-Flight Mass Spectrometry (QTOF-MS) detection of phenolic compounds. Another extraction of the same cocoa powder was done by the same procedure with 20 mL of methanol/H_2_O (50:50 *v*/*v* acidified with formic acid 0.1%).

### 2.3. HPLC-QTOF-MS Analysis of Phenolic Compounds

Analyses were performed using HPLC coupled with a mass spectrometer (HPLC-MS-QTOF). The HPLC (Agilent 1200, Agilent Technologies, Waldrom, Germany) with a quaternary pump (model G1311A) was coupled with a diode array detector (Agilent model G1315B,) and an Agilent 6530 Accurate-Mass QTOF-MS with Electrospray Ionization (ESI) with Jet Stream technology (Agilent Technologies, Santa Clara, CA, USA). Separation was performed on a Phenomenex Luna C18 column (5 μm, 4.6 mm × 150 mm; Phenomenex, Alcobendas, Spain), set thermostatically at 25 °C.

A gradient between solvent A (water/formic acid, 99.9:0.1, *v*/*v*) and solvent B (acetonitrile/formic acid, 99.9:0.1, *v*/*v*) was applied at a flow rate of 0.5 mL/min as follows: 10% B at 0 min, 30% B at 30 min, 35% B at 35 min, 40% B at 45 min, 10% B at 50 min, and 10% at 60 min. The volume of sample injected was 20 μL. The electrospray ionization (ESI) parameters were as follows: drying gas flow, 8 L/min; nebulizer pressure, 45 psi; gas drying temperature, 325 °C; sheath gas temperature, 300 °C; sheath gas flow, 11 L/min; capillary voltage, 4000 kV; and fragmentator, 120 V. The ESI was operated in positive and negative mode to provide extra certainty in the determination of the molecular masses. Quantification was performed in the positive mode by using extracted-ion chromatogram (EIC) data and by comparison with external standard curves. Full data were collected in extended dynamic range, 100−1200 *m*/*z*. For the identification and quantification of compounds, MS and tandem mass spectrometry fragmentation spectra (MS/MS) experiments were performed, and spectral signal at data were also acquired at 280, 320, and 520 nm.

For mass spectrometry experiments, quite generic collision energy of 20 V was used, as a compromise, to simplify development of the method and ensure good fragmentation of the majority of targeted compounds. Data acquisition and processing were performed with a Masshunter Data Acquisition B.05.01 and Masshunter Qualitative Analysis B.07.00 SP2 software (both from Agilent Technologies, Santa Clara, CA, USA). Compounds were identified by comparing mass spectra and retention time with the corresponding standard if available. In the case of compounds for which standards were not available, identification was based on prediction of chemical formula from accurate ion mass measurement and confirmed by comparing MSMS with data provided by relevant literature references (see Section 3.1). The quantification was performed by interpolation into the calibration curve of the standard or structurally related compound used to quantify (equivalent) and expressed as µg per g of dry matter (DM) as follows: epicatechin for oligomeric procyanidins and quercetin-3-*O*-glucoside for flavonols.

### 2.4. Cell Culture and Differentiation

The human cell line SH-SY5Y (neuroblastoma) was maintained in Dulbecco Modified Eagle Medium (DMEM) supplemented with 10% fetal bovine serum (FBS), 1% antibiotics (penicillin/streptomycin 5000 U/mL), and 1% non-essential aminoacids. The SH-SY5Y cell line was kindly provided by Dr. Almeida from the University of Salamanca. Cells were grown in 75 cm^2^ tissue culture flask at 37 °C in a humidified atmosphere containing 5% CO_2_ until 80–90% confluence. Cells were harvested with trypsin-EDTA. SH-SY5Y cells were seeded in 96-well plates for the cytotoxicity test and in 24-well plates for the ROS assay.

Cell differentiation was induced with 10 µM all-*trans* retinoid-acid (RA) (Sigma–Aldrich, St Louis, MO, USA) following the methodology [32,33]. After 24 h, medium was replaced with medium in which the FBS concentration was reduced to 1%, supplemented with 10 μM of RA, and incubated for 5 days. At days 2 and 4, differentiating media was changed, and all the experiments were performed after 5 days of differentiation. Only cells between passages P7 to P15 were used.

### 2.5. Cell Viability Assay

The viability of cells was determined by MTT assay. SH-SY5Y cells were plated in 96-well plates (2 × 10^4^ cells/well), cultured for 24 h at 37 °C in 5% CO_2_ and were differentiated for 5 days with 10 µM RA. The differentiated neurons were treated with serially diluted concentration of epicatechin (epi) or cocoa extract. After 3 and 18 h of incubation, 20 µL of a MTT solution (5 mg/mL in PBS) was added to each well and incubated for an additional 2 h at 37 °C in 5% CO_2_. Formazan crystals formed in the wells were solubilized in 200 µL of Dimethyl sulfoxide (DMSO). Absorbance was measured at 570 nm wavelength employing a microplate reader PowerWaveTM XS (BioTek Instruments, Inc., Winooski, VT, USA). The viability was calculated in comparison to control experiments in which a solvent control was added in place of epicatechin or cocoa extract and that was used as a 100% viable reference [34].

### 2.6. Evaluation of ROS Generation

#### 2.6.1. Fluorimetry in Plated Cells

Cellular ROS were quantified by H2DCFDA assay by using a microplate reader with slight modifications [26]. SH-SY5Y cells were seeded into 24-well plates at a density of 1 × 10^5^ cells/well, and cells were differentiated for 5 days with 10 µM RA. After incubation with cocoa extract (final concentrations: 25, 50, 100, 200 mg/mL dissolved in serum-free DMEM) or EC (final concentrations: 0.5, 1, 10, 50 µM in EtOH/DMSO (1:5 *v*/*v*)) for 3 h, then cells were treated with *tert*-butyl hydroperoxide (*t*-BOOH) (500 µM) for 1h. Total ROS in cells was measured by using 2′,7′-dichlorodihydrofluorescein diacetate (H2DCFDA, Cat D6883, Sigma–Aldrich, St. Louis, MO, USA). H2DCFDA (1 µM) was added to the culture medium and incubated for 30 min. H2DCFDA was metabolized by cell esterases to H2DCF and then oxidized by ROS into the highly fluorescent 2′,7′-dichlorodihydrofluorescein (DCF), and the produced DCF was proportional to ROS generation. For the assay, cells were seeded in 24-well plates at a rate of 2 × 10^5^ cells per well and changed to the different cocoa and EC concentrations the day after. Prior to the end of the assay, 5 µM H2DCFDA was added to the wells for 30 min at 37 °C. Then, cells were washed twice with serum-free medium before multi-well plates were measured in a fluorescent microplate reader at excitation wavelength of 485 nm and emission wavelength of 530 nm [27].

#### 2.6.2. Flow Cytometry

SH-SY5Y cells were seeded into 60 mm plates at a density of 2 × 10^5^ cells/well, and cells were differentiated for 5 days with 10 µM RA. Once differentiated, cells were incubated with the same concentrations of cocoa extract and EC as above for 3 h, treated with *t*-BOOH (500 µM) for 1 h, and 5 µM H2DCFDA was added to the wells for 30 min at 37 °C. Excess H2DCFDA was removed by washing the cells twice with PBS. Labeled cells were trypsinized and resuspended in PBS and then analyzed using a flow cytometer (Cytoflex, Beckman Coulter, Indianapolis, IN, USA). A minimum of 10,000 cells were analyzed per condition. Then, all data were compared to control samples (maximum ROS production) whose fluorescence was considered as 100 [25,28,29,30].

### 2.7. Determination of Reduced Glutathione (GSH) Concentration

The content of GSH was quantitated by the fluorometric assay described in Browne and Armstrong [35], with some modifications. The method takes advantage of the reaction of GSH with *o*-phthalaldehyde (OPT) at pH 8.0; although OPT reacts not only with GSH but also with other thiols, such as methionine, cysteine and *N*-acetylcysteine, comparison to appropriate controls permitted a reliable quantification. After treatment of SH-SY5Y cells with the different concentrations of cocoa extract or EC prior to the oxidative challenge with *t*-BOOH, the culture medium was removed and cells (2 × 10^6^) were detached and homogenized by ultrasound with 5 % trichloroacetic acid containing 2 mM EDTA. Following centrifugation of cells for 30 min at 1000× *g*, 50 µL of the clear supernatant were transferred to a 96-multiwell plate for the assay. Fluorescence was measured at an excitation wavelength of 345 nm and emission wavelength of 425 nm. The results of the samples were referred to those of a standard curve of GSH.

### 2.8. Determination of Glutathione Peroxidase (GPx) and Glutathione Reductase (GR) Activity

For the assay of the GPx and GR activity, treated cells (2 × 10^6^) were suspended in PBS and centrifuged at 300 g for 5 min to pellet cells. Cell pellets were resuspended in 20 mM Tris, 5 mM EDTA, and 0.5 mM mercaptoethanol, sonicated and centrifuged at 3000× *g* for 15 min. Enzyme activities were measured in the supernatants and protein was measured by using the Bradford reagent. Determination of GPx activity is based on the oxidation of GSH by GPx, using *t-*BOOH as a substrate, coupled to the disappearance of Nicotinamide adenine dinucleotide phosphate (NADPH) by GR, as described in Alía et al., [26] with slight modifications. GR activity was determined by following the decrease in absorbance due to the oxidation of NADPH utilized in the reduction of oxidized glutathione [26].

### 2.9. Statistical Analysis

Statistical data analysis was performed though SPSS (Version 27, IBM Corp., Armonk, NY, USA). Data were tested first for normal distribution with the Shapiro–Wilk test and by graphical interpretation using a residual plot. In our analysis, the treatment was regarded as a fixed factor and the replicate as a random factor. Based on this model, an analysis of variances (ANOVA) was conducted, followed by a post-hoc multiple comparison test of Bonferroni to compare the effects to the cell untreated control. Differences were considered significant at *p* < 0.05. All analyses were performed in triplicate, and the results were displayed as average ± standard deviation.

## 3. Results

### 3.1. Chemical Characterization of Cocoa Powdder

The chemical characterization and quantification of cocoa powder is shown in Table 1 and Table 2. Flavan-3-ols and flavonols were the polyphenolic groups identified in cocoa powder and represented 96 and 4%, respectively. Regarding flavan-3-ols, catechin and epicatechin were identified with commercial standard, whereas oligomeric procyanidins were identified based on their molecular masses. Ions from *m*/*z* 291 to 1443 separated by 288 Dalton were identified as the different oligomeric forms. In this sense, four peaks with *m*/*z* 579 (291 + 288) corresponded to procyanidin dimers, five peaks with *m*/*z* 867 (579 + 288) to procyanidin trimers, five peaks with *m*/*z* 1155 (867 + 288) to procyanidin tetramers, and two peaks with *m*/*z* 1443 (1155 + 288) to procyanidin pentamers.

The characterization of flavonols was performed based on the presence of the aglycon quercetin (*m*/*z* 303) derived from the loss of either a glucose (*m*/*z* 162) or an arabinose (*m*/*z* 132) during the MSMS fragmentation. Additionally, the presence of quercetin-3-*O*-glucoside was confirmed using commercial standard.

Three alkaloids, of which two were methylxantines, theobromine (3,7-dimetilxantine), caffeine (1,3,7-trimethylxanthine), and 2,3,5,6-tetramethylpyrazine were identified and confirmed with commercial standards.

**Figure 1:**
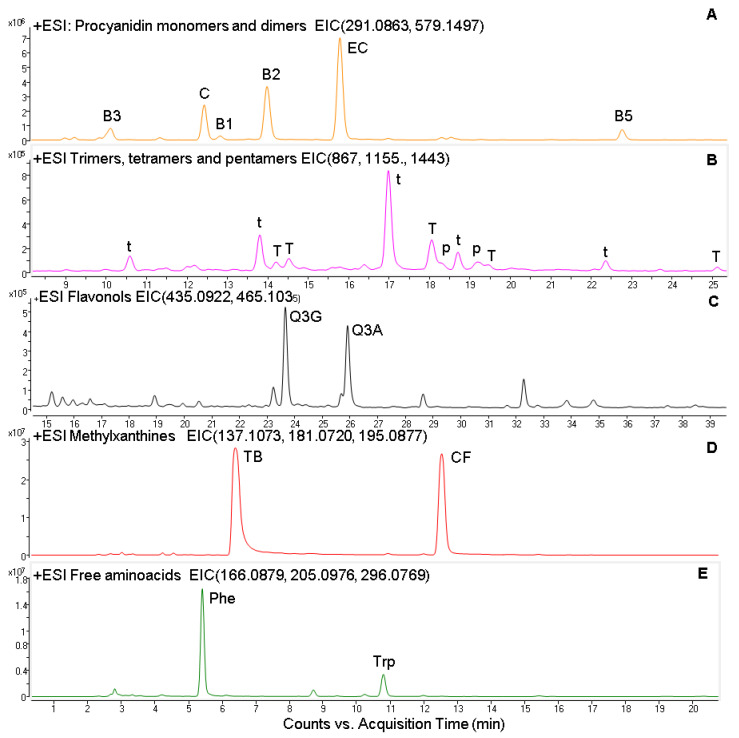
Extracted ion chromatogram (EIC) from the ESI positive analysis of Procyanidin monomers and dimers (**A**); trimers, tetramers, and pentamers (**B**); flavonols (**C**); methylxanthines (**D**) and free aminoacids (**E**). C, catechin; EC, epicatechin, B1, B2, B3, and B5 procyanidin dimers; t, procyanidin trimer; T, procyanidin tetramer; p, procyanidin pentamer; TB, theobromine; CF, caffeine; Phe, l-phenylalanine; Trp, l-tryptophan.

With the chromatographic conditions employed in this study, we identified tryptophan and phenylalanine. The presence of these free amino acids (AAs) containing aromatic groups was confirmed, and these compounds were quantified using commercial standards. Additionally, the presence of a peak with *m*/*z* 296 was identified as caffeoyl aspartic acid. A representative chromatogram is shown in Figure 1.

### 3.2. Cell Viability

In our study, pretreatment of differentiated SH-SY5Y cells with up to 1 mg/mL of cocoa and up to 100 µM of EC did not evoke significant changes on cell viability (data not shown). Thus, it was ruled that none of the effects found were due to a cytotoxic effect.

### 3.3. ROS Generation

A powerful pro-oxidant such as *t*-BOOH can directly decompose to peroxyl radicals and generate lipid peroxides and other ROS, thus increasing fluorescence by binding to dichlorodihydrofluorescein (H2DCFDA) diacetate and generating fluorescent compound DFC. In order to optimize our working conditions, we established the working concentration by performing a dose response curve (data not shown). After exposing the cells to 500 µM *t*-BOOH for 1 h, the maximum amount of ROS is produced, and thus this treatment represents our control for oxidative stress.

**Figure 2:**
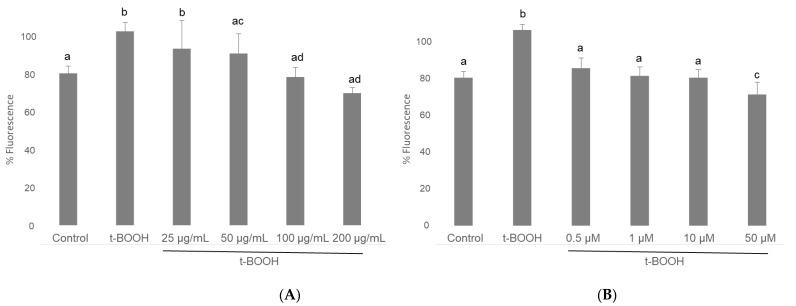
Effect of pre-treatment of differentiated SH-SY5Y cells with cocoa extract (**A**) and EC (**B**) prior to oxidative stress on ROS generation determined by in situ fluorimetry on plated cells. Values are means ± SD, *n* = 8. Values are expressed as a percent relative to control condition. Different letters indicate statistically significant differences (*p* < 0.05) among groups.

Analysis of ROS with a H2DCFDA fluorescent probe on cultured cells in plates and measured in a microplate reader showed that, when neuroblastoma cells differentiated in culture were treated with 500 µM *t*-BOOH for 1 h, the 2-fold increase of intracellular ROS concentration was indicative of a clear situation of oxidative stress (Figure 2). Interestingly, this severe rise of ROS levels was completely prevented when differentiated SH-SY5Y cells were pre-treated with 100 or 200 µg/mL of cocoa extract for 3 h prior to the oxidative challenge (Figure 2); a partial but significant recovery was also observed in cells treated with 25 or 50 µg/mL cocoa. Considering all four conditions, chemo-protective response against ROS increase was dose-dependent. Pretreatment for 3 h of differentiated neuroblastoma cells with EC alone also showed a similar dose-dependent reduction of ROS over-production when cells where submitted to 500 µM *t*-BOOH for 1 h (Figure 2). The doses that showed the highest efficiency for ROS quenching were 100–200 µg/mL cocoa and 1 µM EC.

**Figure 3:**
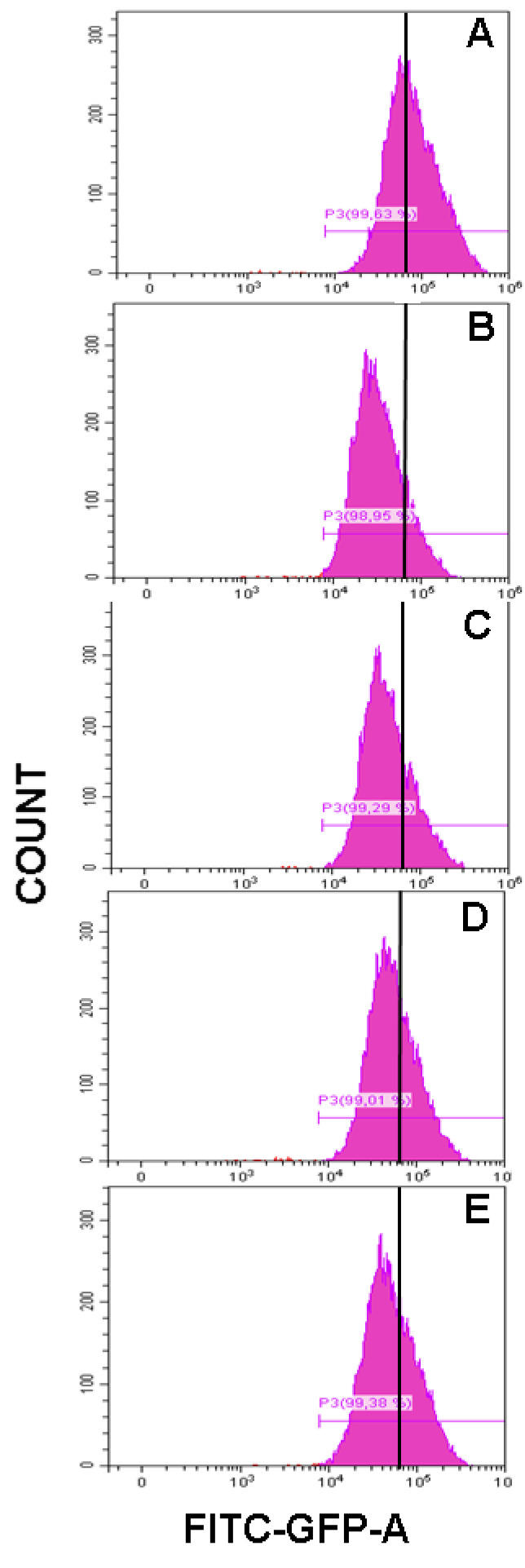
Representative flow-cytometry histograms corresponding to ROS generation in differentiated SH-SY5Y cells for *t*-BOOH treatment (**A**) and *t*-BOOH plus cocoa treatment at different concentrations (**B**, 200 µg/mL; **C**, 100 µg/mL; **D**, 50 µg/mL; and **E**, 25 µg/mL).

**Figure 4:**
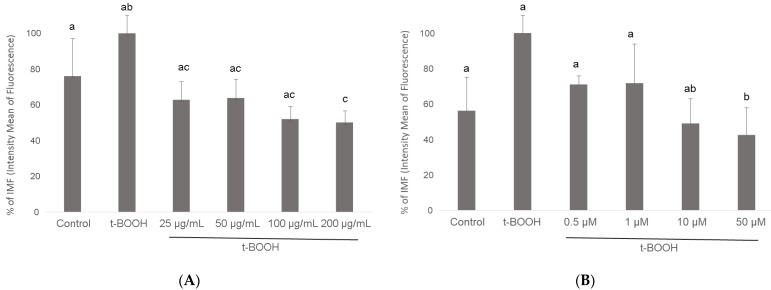
Effect of pre-treatment of differentiated SH-SY5Y cells with cocoa extract (**A**) and EC (**B**) prior to oxidative stress on ROS generation determined by flow cytometry. Values are means ± SD, *n* = 8. Values are expressed as a percent relative to the control condition. Different letters indicate statistically significant differences (*p* < 0.05) among groups.

Flow cytometry histograms showed an increased fluorescence in the FL1-H channel indicative of increased levels of ROS. Thus, the mean fluorescence intensity of the oxidized dichlorofluorescein (DCF) was acutely increased after 500 μM *t*-BOOH treatment in differentiated SH-SY5Y cells, whereas increasing concentration of cocoa extract were able to decrease the signal corresponding to the maximum ROS formation generated by cell treatment with *t*-BOOH (Figure 3). Flow-cytometry assay showed that cocoa inhibited (*p* < 0.05) the ROS production generated by the addition of *t*-BOOH to this SH-SY5Y differentiated cell model. When the cocoa treated cells were compared with the control group, the differentiated cells pre-treated with 200, 100, 50, and 25 µg/mL of cocoa extract reduced (*p* < 0.05) the ROS production by 50%, 52%, 64%, and 63%, respectively (Figure 3 and Figure 4). Regarding EC (Figure 4), a reduction (*p* < 0.05) of ROS production was observed with concentrations of 50 and 10 µM (43% and 49%, respectively, compared to the control group).

### 3.4. GSH Concentration

**Figure 5:**
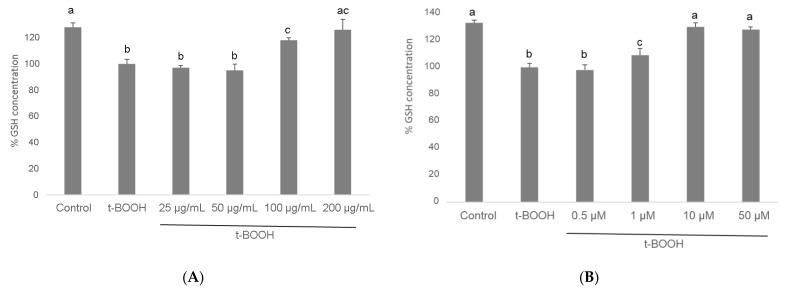
Effect of pre-treatment of differentiated SH-SY5Y cells with cocoa extract (**A**) and EC (**B**) prior to oxidative stress on ROS generation determined by flow cytometry. Values are means ± SD, *n* = 8. Values are expressed as a percent relative to the control condition. Different letters indicate statistically significant differences (*p* < 0.05) among groups.

When differentiated SH-SY5Y cells were submitted to a situation of oxidative stress by the administration of 500 µM *t*-BOOH for 1 h, GSH concentration decreased to around 50% of basal levels (Figure 5). This severe decrease of GSH was partially reversed by a pre-treatment of cells for 3 h with 100 µg/mL of cocoa extract and completely reverted to control values with 200 µg/mL. Similarly, a partial but significant recovery of depleted GSH was observed in differentiated SH-SY5Y cells pre-treated with 1 µM EC for 3 h before the oxidative challenge, whereas pre-treatment with 10 or 50 µM EC evoked a complete recovery of GSH (Figure 5).

These results unequivocally indicate that the presence in the culture media of the antioxidant compounds contained in cocoa extract, or only physiological doses of EC, bestows differentiated SH-SY5Y cells with a significant protection against the loss of reducing power in a situation of oxidative stress. The doses that showed highest efficiency were 200 µg/mL cocoa and 10 µM EC.

### 3.5. GPx Activity

**Figure 6:**
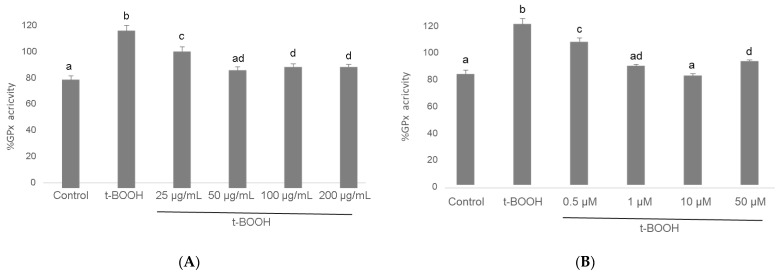
Effect of pre-treatment of differentiated SH-SY5Y cells with cocoa extract (**A**) and EC (**B**) prior to oxidative stress on GPx activity. Values are means ± SD, *n* = 3. Values are expressed as a percent relative to the control condition. Different letters indicate statistically significant differences (*p* < 0.05) among groups.

The challenging of differentiated SH-SY5Y cells with 500 µM *t*-BOOH for 1 h evoked a significant increase in the activity of this antioxidant defense enzyme as a rational response to the severe ROS overproduction (Figure 6). Pre-treatment of neuroblastoma cells with 25–200 µg/mL cocoa extract resulted in a significant recovery of GPx activity after the stress, following a dose-dependent pattern between a partial regain at 25 µg/mL and a whole recuperation at 50 µg/mL, which was the most efficient cocoa concentration (Figure 6). Similarly, pre-treatment of differentiated SH-SY5Y with all four concentrations of EC for 3 h evoked a significant recovery of GPx activity after the stress with 500 µM *t*-BOOH for 1 h. In this case, there was also a dose-dependent response between a limited recovery at 0.5 µM and a complete recovery at 1 and 10 µM EC (Figure 6). The lowest doses that showed the highest efficiency were 50 µg/mL cocoa and 1 µM EC.

### 3.6. GR Activity

**Figure 7:**
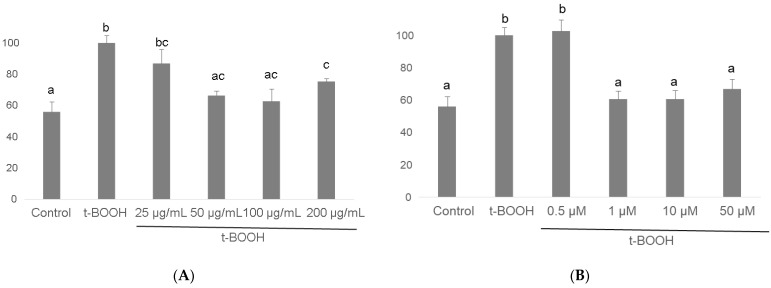
Effect of pre-treatment of differentiated SH-SY5Y cells with cocoa extract (**A**) and EC (**B**) prior to oxidative stress on GR activity. Values are means ± SD, *n* = 3. Values are expressed as a percent relative to the control condition. Different letters indicate statistically significant differences (*p* < 0.05) among groups.

As shown in Figure 7, the *t*-BOOH challenge provoked in differentiated SH-SY5Y a two-fold raise in GR activity to recover the increased oxidized glutathione produced by enhanced GPx activity. As in the case of GPx, pre-treatment for 3 h with all four concentrations of cocoa extract significantly recovered GR activity in a dose-dependent manner between a slight (barely significant) decrease at 25 µg/mL and a through recovery of basal values at 50 and 100 ug/mL, being 200 µg/mL more effective than 25 but less effective than the two lower concentrations (Figure 7). When differentiated neuroblastoma cells were pre-treated with EC for 3 h before the stress with 500 µM *t*-BOOH for 1 h, only the concentrations of 1, 10, and 50 µM evoked a full recovery of the basal GR activity, being the lowest dose of 0.5 µM EC ineffective (Figure 7). As in the case of GPx, the lowest doses that showed the highest efficiency were also 50 µg/mL cocoa and 1 µM EC.

## 4. Discussion

The present study was performed to investigate the potential chemo-protective effect of cocoa polyphenols in cultured neuronal-like cells submitted to an oxidative stress and to elucidate the antioxidant defense mechanisms involved. Furthermore, two different methods to evaluate ROS generation in stressed conditions were used in differentiated neuroblastoma cells and the results compared between them and related to other markers of antioxidant defense. The results demonstrate for the first time that an aqueous polyphenolic extract from cocoa powder and its main monomeric flavanol, EC, have the capacity to protect differentiated human neuroblastoma cells against an oxidative insult by modulating oxygen radical generation and enzyme and non-enzyme antioxidant defenses. Besides, both methods tested for ROS generation showed consistent data confirming that H2DCFDA assay can be used as reliable methodology, both by microplate reader on plated cells and by flow cytometry in cultured differentiated neuroblastoma cells.

Biological activities of cocoa flavanols include antioxidant capacity, anti-inflammatory activity, prevention of LDL oxidation, regulation of apoptotic and survival/proliferation pathways, and cardiovascular benefits; all these properties may also exert neuroprotective functions, helping to improve cognitive function and memory capacity, predominantly among older populations at risk or with cognitive decline [3,12,13,15,16,17,18]. Recent revisions have confirmed a positive role for cocoa flavonoids on cerebral blood flow [3,12,18], and a very recent systematic review [36] has reported that memory and executive function can increase significantly after intake of realistic doses of cocoa flavanols (500–750 mg/day). Although all these data make cocoa polyphenolic fraction an interesting candidate for neurovascular chemo-protection, the beneficial effects of this phenolic fraction and specific flavanols on neuronal-like cells need to be elucidated. Cocoa and different enantiomers of catechin and epicatechin have shown similar protective effects in undifferentiated SH-SY5Y neuroblastoma cell lines [24,37,38]; however, these effects have not been previously tested in a more reliable neuronal-like model such as differentiated SH-SY5Y [25,31]. Thus, in the present study, the effects of a cocoa phenolic extract and its main flavonoid component, EC, on the antioxidant defense system of differentiated neuroblastoma cells in response to an induced oxidative stress have been studied for the first time.

Cocoa beans are rich in polyphenols, in particular flavan-3-ols, showing a variable content according to the variety and growing environments, but also to the conditions employed during their fermentation and drying processes [39,40]. In the extract prepared for the present study, total flavan-3-ols represented 96% of the total polyphenols, monomeric EC and catechin being the most abundant structures (56%), followed by dimeric (32%), trimeric (9%), tetrameric (2.8%), and pentameric procyanidins (0.5%). The solvent used during extraction affects the yield and mean degree of polymerization of the extracted proanthocyanidins [41], water being the less effective for polymerized procyanidins. Accordingly, methanolic extraction (50:50) yielded a higher content of oligomeric structures than water extraction. However, in the present study we decided to use a water extract because the final aim was to determine the effect of cocoa on neuronal viability and redox response in conditions that could mimic, at least partially, the physiological ones. Regarding flavonols, this group represented 4% of total polyphenol, where similar contents of quercetin-3-*O*-arabinoside and quercetin-3-*O*-glucoside were obtained.

Apart from polyphenols, cocoa products are also known to contain alkaloids such as theobromine and caffeine [42,43]. As a whole, methylxanthines (theobromine being the predominant compound) were found in higher concentration than polyphenols. These compounds contribute to the bitterness of the cocoa [43]. Additionally, in this extract we identified the presence of tetrametylpyrazine, a pyrazine formed during the roasting and fermentation processes of cocoa, representing about 40% of the compound identified in the cocoa-aroma fraction [44]. Proteins contribute to 10–15% of cocoa seeds, mainly consisting of albumin, globulin, and fractions, whereas the non-protein free amino acids represent a lower amount (around 0.3%) but also play an important role on cocoa aroma [45]. Free amino acids are cocoa aroma precursors formed and affected by the fermentation and drying processes [46]. Triptophan is a precursor in the synthesis of the neurotransmitter serotonin, which is involved in the regulation of appetite, circadian rhythm, and affective reaction control because it is absorbable and able to cross the blood brain barrier [47]. A significant amount of caffeoyl aspartic acid was also identified in this cocoa extract. The presence of this and other hydroxycinnamoyl amino acid conjugates has also been previously reported in cocoa bean [48,49].

Thus, liquid chromatography-mass spectrometry analysis confirmed that the cocoa aqueous phenolic extract used in this study is a realistic representative of cocoa-derived products. In fact, composition of our cocoa extract is very similar to other cocoa phenolic extracts previously prepared and tested in the literature [50,51,52]. Concerning the concentrations to be tested, authors have reported concentrations in the range of 0.2–0.4 µM EC after consumption of 50 g of chocolate [53,54] and others have observed up to 35 µM of EC in rat serum 1 h after oral administration of EC [55]. In this context, 30 µM EC showed a chemo-protective effect in undifferentiated neuroblastoma SH-SY5Y submitted to an oxidative stress [24]. Similarly, 10 µM EC proved to be effective in colonic cells Caco-2 submitted to a similar oxidative challenge [56], and 20 µM EC was shown to protect pancreatic beta cells INS-1E against *t*-BOOH-induced oxidative stress [57]. Regarding the cocoa extract, a significant protection of undifferentiated neuroblastoma cells was reported with 30 µg/mL [24], as well as with 50 µg/mL on cultured hepatic cells [50,51], 20 µg/mL in pancreatic beta cells INS-1E [58], and the same concentration in cultured endothelial cells [59]. Because these ranges of low supra-physiological concentrations of cocoa extract and EC showed a chemo-protective effect against oxidative stress in different cell lines, a comparable range of concentrations was assayed in differentiated neuroblastoma cells, as a neuronal-like cell culture model, and their response to oxidative stress conditions was tested. Although elevated doses of dietary antioxidants may also act as pro-oxidants in cell culture systems and evoke cellular damage [60], cytotoxicity was assayed with all doses tested in this study and none of them caused significant cell damage.

Cocoa flavonoids are effective scavengers of oxygen radicals in cultured cells [24,50], and a decreased production of ROS might reveal a reduced intracellular oxidation and a balanced redox status that represents an advantageous condition for the cell to face a potential oxidative insult. Fluorescent measurement of ROS by dichlorofluorescein diacetate probe in situ on cultured cells has been widely used as a reliable test [26,27], but some authors consider that this method is not very specific and in some cases does not take into consideration the cytotoxicity, while flow cytometry has been proven to be a more reproducible alternative to measure ROS generation [25,30]. In this study, both methods were used to evaluate ROS production in differentiated SH-SY5Y submitted to an oxidative stress and pretreated with cocoa extract and EC. When cellular ROS generation was enhanced by a potent pro-oxidant such as *t*-BOOH on differentiated SH-SY5Y cells, a significant increase of ROS levels was observed, both in plated cells in situ measured in a microplate reader as well as in cells scraped and counted in a flow cytometer. Moreover, a significant dose-dependent reduction of ROS in differentiated SH-SY5Y cells that were pre-treated with cocoa extract or EC was detected by both assayed methods. A similar ROS quenching effect has been previously reported for cocoa extracts and specific flavanols in other cell lines [50,56,58] and in undifferentiated SH-SY5Y [24]. These results suggest that the ROS generated during the period of oxidative stress were more efficiently quenched in differentiated SH-SY5Y cells treated with cocoa extract or EC, surely reducing any ROS-induced cell damage.

GSH, the main non-enzymatic antioxidant defense in the cell, acts as a substrate in GPx-catalysed detoxification of organic peroxides, reacts with free radicals, and repairs free radical induced damage through electron-transfer reactions. Because depletion of cellular GSH plays a critical role in apoptotic signaling [61], preserving GSH concentration above a critical threshold while facing a stressful insult represents a significant benefit for cell survival. The dose-dependent recovery of GSH concentration when differentiated SH-SY5Y cells were pre-treated with either cocoa extract or EC clearly indicates an efficient cellular chemo-protection against the oxidative challenge. As in the case of ROS levels, a chemo-protective effect of cocoa flavanols on GSH concentration has been previously reported in other cell lines [50,56,58,59]. However, this is the first time that a protective effect on GSH has been shown by a plant extract or any specific compound in differentiated neuroblastoma cells.

Enhancement of glutathione-dependent enzymes GPx and GR are critical mechanisms of the cell defense system to face ROS overproduction and prevent their cytotoxicity [26,27,51]. GPx activity quenches ROS at the expense of GSH, which becomes oxidized and is recovered again to GSH by GR activity. However, a rapid return of the antioxidant enzyme activities to basal values once the challenge is overcome will set an advantageous condition for the cell to deal with a new oxidative insult. Thus, pre-treatment of differentiated SH-SY5Y cells with cocoa extract or pure EC partially or completely prevented the enduring raise in the activities of GPx and GR induced by oxidative stress, returning the antioxidant defenses to values that were significantly closer to basal activity, which guaranteed that the cells were in better conditions to survive further oxidative challenges. Again, although a similar antioxidant defense response has been previously reported for cocoa flavonoids in different cell types [50,56,58,59], this essential defense mechanism for chemo-protection has never been described for differentiated neuroblastoma cells submitted to an oxidative stress.

## 5. Conclusions

In conclusion, the results validate both assays to evaluate ROS concentration, in situ fluorimetry and flow cytometry, in differentiated neuroblastoma submitted to oxidative stress. Overall, the results indicate that differentiated neuroblastoma cells in culture are protected against an oxidative stress by a pre-treatment with physiological or low-supra-physiological concentrations of an aqueous cocoa phenolic extract and its main flavonoid compound, EC. In particular, concentrations of 100–200 µg/mL cocoa extract are necessary to recover ROS and GSH from a severe oxidative stress induced by *t*-BOOH in differentiated SH-SY5Y, whereas 50 µg/mL cocoa is enough to recuperate the activity of antioxidant enzymes. In the case of EC, 1 µM was sufficient to normalize ROS values and GPx/GR while at least 10 µM EC is needed to normalize GSH. These data suggest that the chemo-protective effect of cocoa extract and EC in differentiated neuroblastoma cells could help design or change dietary patterns or nutritional recommendations in order to prevent or delay cognitive decline, dementia, and other neurodegenerative diseases where oxidative stress has been recognized as a risk factor.

## Figures and Tables

**Table 1:** Chemical characterization of cocoa powder.

Compound Assignement	RT^1^(minn)	Molecular Formula	[M + H]^+^ Theoretical	[M + H]^+^ Identified	MSMS
Flavan-3-ols					
*Monomers*					
Catechin	12.5	C_15_H_14_O_6_	291.0863	291.0875	139, 123
Epicatechin	15.8	C_15_H_14_O_6_	291.0863	291.0875	139, 123, 147
*Dimers*					
Procyanidin B3	10.1	C_30_H_26_O_12_	579.1497	579.1524	289, 127, 409
Procyanidin B1	12.9	C_30_H_26_O_12_	579.1497	579.2232	409, 275, 127
Procyanidin B2	13.9	C_30_H_26_O_12_	579.1497	579.1451	127, 291, 409
Procyanidin B5	22.8	C_30_H_26_O_12_	579.1497	579.1503	409, 291, 127
*Trimers*					
Procyanidin trimer (CCC)	10.6	C_45_H_38_O_18_	867.2131	867.2139	
Procyanidin trimer (EEC)	13.8	C_45_H_38_O_18_	867.2131	867.2129	579, 289, 451, 247, 697
Procyanidin trimer (EEE)	16.9	C_45_H_38_O_18_	867.2131	867.2039	579, 289, 427, 247, 139
Procyanidin trimer	18.7	C_45_H_38_O_18_	867.2131	867.2129	580, 291, 425, 163
Procyanidin trimer	22.4	C_45_H_38_O_18_	867.2131	867.2149	
*Tetramers*					
Procyanidin tetramer	14.2	C_60_H_50_O_24_	1155	1155.2749	
Procyanidin tetramer	14.6	C_60_H_50_O_24_	1155	1155.2762	
Procyanidin tetramer (EEEE)	18.2	C_60_H_50_O_24_	1155	1155.2711	579, 867, 289
Procyanidin tetramer	19.45	C_60_H_50_O_24_	1155	1155.2756	
Procyanidin tetramer	25.3	C_60_H_50_O_24_	1155	1155.2761	
*Pentamers*					
Procyanidin pentamer	18.5	C_75_H_62_O_30_	1443	1443.3423	
Procyanidin pentamer	19.3	C_75_H_62_O_30_	1443	1443.3391	
Flavonols					
Quercetin-3-*O*-glucoside	23.6	C_21_H_20_O_12_	465.1035	465.1932	303
Quercetin-3-*O*-arabinoside	25.9	C_20_H_18_O_11_	435.0922	435.0928	303
Free aminoacids					
Caffeoyl aspartic acid	10.9	C_13_H_13_NO_7_	296.0768	296.0769	163
L-phenylalanine	5.22	C_9_H_11_NO_2_	166.0879	166.0858	120
l-tryptophan	8.2	C_11_H_12_N_2_O_2_	205.0976	205.0979	146, 118
Methylxanthines					
Theobromine	6.37	C_7_H_8_N_4_O_2_	181.0720	181.0718	138, 110, 163
Caffeine	12.6	C_8_H_10_N_4_O_2_	195.0877	195.0879	138, 110, 83
Tetramethylpyrazine	10.9	C_8_H_12_N_2_	137.1073	137.1074	96, 55

**Table 2:** Composition of the cocoa powder extracted with water or methanol:water (50:50).

Compound, µg/g *	Water Extract	MetOH Extract
Flavan-3-ols		
*Monomers*		
Catechin	462 ± 5,6	401 ± 16.9
Epicatechin	1867 ± 32.3	1322 ± 24.5
*Dimers*		
Procyanidin B3	143 ± 16.2	118 ± 5.6
Procyanidin B1	59.5 ± 5.1	45.2 ± 3.2
Procyanidin B2	870 ± 52.0	701 ± 30.3
Procyanidin B5	125 ± 28.9	119 ± 4.6
*Trimers*		
Procyanidin trimer	18.0 ± 4.1	23.5 ± 2.3
Procyanidin trimer	50.8 ± 13.3	55.2 ± 3.6
Procyanidin trimer (EEE)	135 ± 28.7	164 ± 10.0
Procyanidin trimer	21.9 ± 6.3	27.8 ± 1.2
Procyanidin trimer	13.2 ± 4.0	14.9 ± 1.1
*Tetramers*		
Procyanidin tetramer	9.0 ± 2.1	11.9 ± 1.0
Procyanidin tetramer	11.0 ± 2.6	16.4 ± 1.4
Procyanidin tetramer	29.2 ± 8.1	42.9 ± 3.8
Procyanidin tetramer	6.9 ± 0.9	8.56 ± 1.7
Procyanidin tetramer	4.3 ± 1.1	6.0 ± 0.5
*Pentamers*		
Procyanidin pentamer	3.2 ± 0.8	4.7 ± 0.2
Procyanidin pentamer	6.7 ± 1.4	11.0 ± 1.1
Flavonols		
Quercetin-3-*O*-glucoside	98.7 ± 9.7	68.4 ± 3.8
Quercetin-3-*O*-arabinoside	71.6 ± 9.1	63.2 ± 4.1
Free amino acids		
l-phenylalanine	424 ± 15.3	240 ± 1.8
l-tryptophan	99.9 ± 2.0	41.0 ± 0.6
*Methylxanthines*		
Theobromine	6684 ± 41.2	5713 ± 19.6
Caffeine	2754 ± 115	2599 ± 51.1
*Tetramethylpyrazine*	1.97 ± 0.1	1.49 ± 0.1

* Catechin, epicatechin, quercetin-3-*O*-glucoside, l-phenylalanine, l-tryptophan, theobromine, and caffeine were quantified by interpolation of their EIC areas in the standard curves constructed with comitial standards. All other procyanidins were interpolated in the epicatechin curve, and quercetin-3-*O*-arabinoside in that of quercetin-3-*O*-glucoside.

## Data Availability

Data is contained within the article.

## References

[1] Murman D.L. (2015). The Impact of Age on Cognition. Semin. Hear..

[2] Livingston G., Sommerlad A., Orgeta V., Costafreda S.G., Huntley J., Ames D., Ballard C., Banerjee S., Burns A., Cohen-Mansfield J. (2017). Dementia prevention, intervention, and care. Lancet.

[3] Cichon N., Saluk-Bijak J., Gorniak L., Przyslo L., Bijak M. (2020). Flavonoids as a natural enhancer of neuroplasticity. An overview of the mechanism of neurorestorative action. Antioxidants.

[4] Moore K., Hughes C.F., Ward M., Hoey L., McNulty H. (2018). Diet, nutrition and the ageing brain: Current evidence and new directions. Proc. Nutr. Soc..

[5] Flanagan E., Lamport D., Brennan L., Burnet P., Calabrese V., Cunnane S.C., de Wilde M.C., Dye L., Farrimond J.A., Emerson Lombardo N. (2020). Nutrition and the ageing brain: Moving towards clinical applications. Ageing Res. Rev..

[6] Vinciguerra F., Graziano M., Hagnäs M., Frittitta L., Tumminia A. (2020). Influence of the Mediterranean and Ketogenic Diets on Cognitive Status and Decline: A Narrative Review. Nutrients.

[7] Vauzour D., Camprubi-Robles M., Miquel-Kergoat S., Andres-Lacueva C., Bánáti D., Barberger-Gateau P., Bowman G.L., Caberlotto L., Clarke R., Hogervorst E. (2017). Nutrition for the ageing brain: Towards evidence for an optimal diet. Ageing Res. Rev..

[8] Dominguez L.J., Barbagallo M. (2018). Nutritional prevention of cognitive decline and dementia. Acta Biomed..

[9] Bell L., Lamport D.J., Butler L.T., Williams C.M. (2015). A review of the cognitive effects observed in humans following acute supplementation with flavonoids, and their associated mechanisms of action. Nutrients.

[10] Carrillo J.A., Zafrilla M.P., Marhuenda J. (2019). Cognitive Function and Consumption of Fruit and Vegetable Polyphenols in a Young Population: Is There a Relationship?. Foods.

[11] Rajaram S., Jones J., Lee G.J. (2019). Plant-Based Dietary Patterns, Plant Foods, and Age-Related Cognitive Decline. Adv. Nutr..

[12] Ammar A., Trabelsi K., Müller P., Bouaziz B., Boukhris O., Glenn J.M., Bott N., Driss T., Chtourou H., Müller N. (2020). The Effect of (Poly)phenol-Rich Interventions on Cognitive Functions and Neuroprotective Measures in Healthy Aging Adults: A Systematic Review and Meta-Analysis. J. Clin. Med..

[13] Di Meo F., Valentino A., Petillo O., Peluso G., Filosa S., Crispi S. (2020). Bioactive Polyphenols and Neuromodulation: Molecular Mechanisms in Neurodegeneration. Int. J. Mol. Sci..

[14] Sánchez-Rabaneda F., Jáuregui O., Casals I., Andrés-Lacueva C., Izquierdo-Pulido M., Lamuela-Raventos R.M. (2003). Liquid chromatographic/electrospray ionization tandem mass spectrometric study of the phenolic composition of cocoa (Theobroma cacao). J. Mass Spectrom..

[15] Spencer J.P.E. (2010). The impact of fruit flavonoids on memory and cognition. Brit. J. Nutr..

[16] Socci V., Tempesta D., Desideri G., De Gennaro L., Ferrara M. (2017). Enhancing human cognition with cocoa flavonoids. Front. Nutr..

[17] Haskell-Ramsay C.F., Schmitt J., Actis-Goretta L. (2018). The impact of epicatechin on human cognition: The role of cerebral blood flow. Nutrients.

[18] Martín M.A., Goya L., de Pascual-Teresa S. (2020). Effect of Cocoa and Cocoa Products on Cognitive Performance in Young Adults. Nutrients.

[19] Drouin A., Bolduc V., Thorin-Trescases N., Bélanger E., Fernandes P., Baraghis E., Lesage F., Gillis M.A., Villeneuve L., Hamel E. (2011). Catechin treatment improves cerebrovascular flow-mediated dilation and learning abilities in atherosclerotic mice. Am. J. Physiol. Heart Circ. Physiol..

[20] Nehlig A. (2013). The neuroprotective effects of cocoa flavanol and its influence on cognitive performance. Br. J. Clin. Pharmacol..

[21] Grassi D., Socci V., Tempesta D., Ferri C., De Gennaro L., Desideri G., Michele F. (2016). Flavanol-rich chocolate acutely improves arterial function and working memory performance counteracting the effects of sleep deprivation in healthy individuals. J. Hypertens..

[22] Faria A., Pestana D., Teixeira D., Couraud P.O., Romero I., Weksler B., De Freitas V., Mateus N., Conceição C. (2011). Insights into the putative catechin and epicatechin transport across blood-brain barrier. Food Funct..

[23] Figueira I., Menezes R., Macedo D., Costa I., Dos Santos C.N. (2017). Polyphenols Beyond Barriers: A Glimpse into the Brain. Curr. Neuropharmacol..

[24] Ramiro-Puig E., Casadesús G., Lee H.G., Zhu X., McShea A., Perry G., Pérez-Cano F.J., Smith M.A., Castell M. (2009). Neuroprotective effect of cocoa flavonoids on in vitro oxidative stress. Eur. J. Nutr..

[25] Carballeda-Sangiao N., Chamorro S., de Pascual-Teresa S. (2021). A red-berry mixture as a nutraceutical: Detailed composition and neuronal protective effect. Molecules.

[26] Alia M., Ramos S., Mateos R., Granado-Serrano A.B., Bravo L., Goya L. (2006). Quercetin protects human hepatoma HepG2 against oxidative stress induced by tert-butyl hydroperoxide. Toxicol. Appl. Pharmacol..

[27] Goya L., Martín M.A., Ramos S., Mateos R., Bravo L. (2009). A cell culture model for the assessment of the chemopreventive potential of antioxidant compounds. Curr. Nutr. Food Sci..

[28] Woolley J.F., Stanicka J., Cotter T.G. (2013). Recent advances in reactive oxygen species measurement in biological systems. Trends Biochem. Sci..

[29] Cásedas G., González-Burgos E., Smith C., López V., Gómez-Serranillos M.P. (2018). Regulation of redox status in neuronal SH-SY5Y cells by blueberry (*Vaccinium myrtillus* L.) juice, cranberry (*Vaccinium macrocarpon* A.) juice and cyanidin. Food Chem. Toxicol..

[30] Shehat M.G., Tigno-Aranjuez J. (2019). Flow Cytometric Measurement of ROS Production in Macrophages in Response to FcγR Cross-linking. J. Vis. Exp..

[31] Shipley M.M., Mangold C.A., Szpara M.L. (2016). Differentiation of the SH-SY5Y Human Neuroblastoma Cell Line. J. Vis. Exp..

[32] Cheung Y.T., Lau W.K., Yu M.S., Lai C.S., Yeung S.C., So K.F., Chang R.C. (2009). Effects of all-trans-retinoic acid on human SH-SY5Y neuroblastoma as in vitro model in neurotoxicity research. Neurotoxicology.

[33] de Bittencourt Pasquali M.A., de Ramos V.M., Albanus R.D.O., Kunzler A., de Souza L.H.T., Dalmolin R.J.S., Gelain D.P., Ribeiro L., Carro L., Moreira J.C.F. (2016). Gene Expression Profile of NF-κB, Nrf2, Glycolytic, and p53 Pathways During the SH-SY5Y Neuronal Differentiation Mediated by Retinoic Acid. Mol. Neurobiol..

[34] Yew M.Y., Koh R.Y., Chye S.M., Othman I., Ng K.Y. (2014). Edible bird’s nest ameliorates oxidative stress-induced apoptosis in SH-SY5Y human neuroblastoma cells. BMC Complement. Altern. Med..

[35] Browne R.W., Armstrong D. (1998). Reduced glutathione and glutathione disulfide. Methods Mol. Biol..

[36] Barrera-Reyes P.K., de Lara J.C., González-Soto M., Tejero M.E. (2020). Effects of Cocoa-Derived Polyphenols on Cognitive Function in Humans. Systematic Review and Analysis of Methodological Aspects. Plant. Foods Hum. Nutr..

[37] Ruan H.L., Yang Y., Zhu X.N., Wang X.L., Chen R.Z. (2011). Similar potency of catechin and its enantiomers in alleviating 1-methyl-4-phenylpyridinium ion cytotoxicity in SH-SY5Y cells. J. Pharm. Pharmacol..

[38] Meireles M., Moura E., Vieira-Coelho M.A., Santos-Buelga C., Gonzalez-Manzano S., Dueñas M., Mateus N., Faria A., Calhau C. (2016). Flavonoids as dopaminergic neuromodulators. Mol. Nutr. Food Res..

[39] Natsume M., Osakabe N., Yamagishi M., Takizawa T., Nakamura T., Miyatake H., Hatano T., Yoshida T. (2000). Analyses of polyphenols in cacao liquor, cocoa, and chocolate by normal-phase and reversed-phase HPLC. Biosci. Biotechnol. Biochem..

[40] Wollgast J., Pallaroni L., Agazzi M.E., Anklam E. (2001). Analysis of procyanidins in chocolate by reversed-phase high-performance liquid chromatography with electrospray ionisation mass spectrometric and tandem mass spectrometric detection. J. Chromatogr. A.

[41] Bosso A., Guaita M., Petrozziello M. (2016). Influence of solvents on the composition of condensed tannins in grape pomace seed extracts. Food Chem..

[42] Brunetto M.R., Gutiérrez L., Delgado Y., Gallignani M., Zambrano A., Gómez A., Ramos G., Romero C. (2007). Determination of theobromine, theophylline and caffeine in cocoa samples by high-performance liquid chromatographic method with on-line cleanup in a switching-column system. Food Chem..

[43] Matissek R. (1997). Evaluation of xanthine derivatives in chocolate—Nutritional and chemical aspects. Z. Lebensm. Unters. Forsch..

[44] Maga J.A. (1992). Pyrazine update. Food Rev. Int..

[45] Zak D.L., Keeney P.G. (1976). Changes in cocoa proteins during ripening of fruit, fermentation, and further processing of cocoa beans. J. Agric. Food Chem..

[46] Rhosius C., Matissek R., Lieberei R. (2006). Free amino acids amounts in raw cocoa from different origins. Eur. Food Res. Technol..

[47] Pardridge W.M. (1979). The role of blood-brain barrier transport of tryptophan and other neutral amino acids in the regulation of substrate-limited pathways of brain amino acid metabolism. J. Neural. Transm. Suppl..

[48] Tomas-Barberan F.A., Cienfuegos-Jovellanos E., Marín A., Muguerza B., Gil-Izquierdo A., Cerda B., Zafrilla P., Morillas J., Mulero J., Ibarra A. (2007). A new process to develop a cocoa powder with higher flavonoid monomer content and enhanced bioavailability in healthy humans. J. Agric. Food Chem..

[49] Febrianto N.A., Zhu F. (2020). Changes in the Composition of Methylxanthines, Polyphenols, and Volatiles and Sensory Profiles of Cocoa Beans from the Sul 1 Genotype Affected by Fermentation. J. Agric. Food Chem..

[50] Martín M.A., Ramos S., Mateos R., Granado-Serrano A.B., Izquierdo-Pulido M., Bravo L., Goya L. (2008). Protection of human HepG2 cells against oxidative stress by cocoa phenolic extract. J. Agric. Food Chem..

[51] Martín M.A., Granado-Serrano A.B., Ramos S., Izquierdo-Pulido M., Bravo L., Goya L. (2010). Cocoa flavonoids up-regulate antioxidant enzymes activity via ERK1/2 pathway to protect against oxidative stress-induced apoptosis in HepG2 cells. J. Nutr. Biochem..

[52] Cordero-Herrera I., Martín M.A., Goya L., Ramos S. (2015). Cocoa flavonoids protect hepatic cells against high glucose-induced oxidative stress: Relevance of MAPKs. Mol. Nutr. Food Res..

[53] Wang J.F., Schramm D.D., Holt R.R., Ensunsa J.L., Fraga C.G., Schmitz H.H., Keen C.L. (2000). A dose response effect from chocolate consumption on plasma epicatechin and oxidative damage. J. Nutr..

[54] Rein D., Lotito S., Holt R.R., Keen C.L., Schmitz H.H., Fraga C.G. (2000). Epicatechin in human plasma: In vivo determination and effect of chocolate consumption on plasma oxidation status. J. Nutr..

[55] Baba S., Osakabe N., Natsume N., Muto Y., Takizawa T., Terao J. (2001). In vivo comparison of the bioavailability of catechin, epicatechin and their mixture in orally administered rats. J. Nutr..

[56] Rodríguez-Ramiro I., Martín M.A., Ramos S., Bravo L., Goya L. (2011). Comparative effects of dietary flavanols on antioxidant defenses and their response to oxidant-induced stress in Caco2 cells. Eur. J. Nutr..

[57] Martín M.A., Fernández-Millán E., Ramos S., Bravo L., Goya L. (2014). Cocoa flavonoid epicatechin protects pancreatic beta cell viability and function against oxidative stress. Mol. Nutr. Food Res..

[58] Martin M.A., Ramos S., Cordero-Herrero I., Bravo L., Goya L. (2013). Cocoa phenolic extract protects pancreatic beta cells against oxidative stress. Nutrients.

[59] Martins T.F., Palomino O.M., Alvarez-Cilleros D., Martin M.A., Ramos S., Goya L. (2020). Cocoa Flavanols Protect Human Endothelial Cells from Oxidative Stress. Plant. Foods Hum. Nutr..

[60] Azam S., Hadi N., Khan N.U., Hadi S.M. (2004). Prooxidant property of green tea polyphenols epicatechin and epigallocatechin-3-gallate: Implications for anticancer properties. Toxicol. In Vitro.

[61] Ramos S., Rodriguez-Ramiro I., Martin M.A., Goya L., Bravo L. (2011). Dietary flavanols exert different effects on antioxidant defenses and apoptosis/proliferation in Caco-2 and SW480 colon cancer cells. Toxicol. In Vitro.

